# Perceptual assessment of environmental stability modulates postural sway

**DOI:** 10.1371/journal.pone.0206218

**Published:** 2018-11-09

**Authors:** Natalia Cooper, Iain Cant, Mark D. White, Georg F. Meyer

**Affiliations:** 1 Construction Research Centre, National Research Council Canada, Ottawa, ON, Canada; 2 Virtual Engineering Centre, Hartree Centre Sci-Tech Daresbury, Keckwick Lane, Daresbury, Warrington, United Kingdom; 3 School of Engineering, Brownlow Hill, University of Liverpool, Liverpool, United Kingdom; 4 Psychological Sciences, University of Liverpool, United Kingdom; University of Melbourne, AUSTRALIA

## Abstract

We actively maintain postural equilibrium in everyday life, and, although we are unaware of the underlying processing, there is increasing evidence for cortical involvement in this postural control. Converging evidence shows that we make appropriate use of ‘postural anchors’, for example static objects in the environment, to stabilise our posture. Visually evoked postural responses (VEPR) that are caused when we counteract the illusory perception of self-motion in space (vection) are modulated in the presence of postural anchors and therefore provide a convenient behavioural measure. The aim of this study is to evaluate the factors influencing visual appraisal of the suitability of postural anchors. We are specifically interested in the effect of perceived ‘reality’ in VR the expected ‘stability’ of visual anchors. To explore the effect of ‘reality’ we introduced an accommodation-vergence conflict. We show that VEPR are appropriately modulated only when virtual visual ‘anchors’ are rendered such that vergence and accommodation cues are consistent. In a second experiment we directly test whether cognitive assessment of the likely stability of real perceptual anchors (we contrast a ‘teapot on a stand’ and a ‘helium balloon’) affects VEPR. We show that the perceived positional stability of environmental anchors modulate postural responses. Our results confirm previous findings showing that postural sway is modulated by the configuration of the environment and further show that an assessment of the stability and reality of the environment plays an important role in this process. On this basis we propose design guidelines for VR systems, in particular we argue that accommodation-vergence conflicts should be minimised and that high quality motion tracking and rendering are essential for high fidelity VR.

## Introduction

It is generally accepted that human bipedal upright stance is achieved by feedback mechanisms that generate appropriate actions to correct for natural body-sway motion detected by our sensory systems [1] [2]. While spinal and brainstem circuits have long been established as fast (Sherrington, 1910) and pre-attentive (Magnus, 1926) postural control mechanisms, there is increasing evidence of significant cortical involvement in modulating postural responses [3, 4].

Visual information, including peripheral motion perception [5], provides the dominant input to postural stability during quiet, unperturbed stance, because of the relatively high sensitivity of the visual motion detection system compared with the proprioceptive and vestibular inputs. Balance is maintained when vision is denied, but body sway typically doubles in amplitude [6].

Externally generated visual motion, which also causes the perception of illusory self-motion (vection), typically leads to a postural response to counteract the perceived motion. These visually evoked postural responses (VEPR) have been documented under a range of conditions [6–11] and depend on stimulus characteristics such as motion speed [12], stimulus texture [13] or position of the stimulus within the visual field [8, 14]. Guerraz et al. [15] argue that, in order to induce consistent and directionally specific responses, the relative motion of objects in the environment must reflect the motion signals that a moving observer would experience in a stable 3-D environment.

These findings are consistent with the view that automatic responses to optokinetic stimulation make an important contribution to postural control [16].

Other data shows that postural responses are modulated by a range of factors that preclude fully automatic processing, such as the configuration of the environment [11, 17–19], expectation [20, 21], and competing cognitive demands, particularly in ageing [22]. One of the key cues controlling VEPRs for static [15] and moving [23] observers is motion parallax, the differential motion of objects in the environment. Simple, automatic mechanisms, for example based on optic flow signals that are integrated over large areas to counteract self-motion, cannot explain the dominant compensatory actions in response to observed parallax motion [15, 23].

VEPRs are therefore likely to be mediated by different mechanisms with different degrees of automaticity and cortical involvement: Guerraz and colleages [24], for example propose a short latency system, driven by transient visual stimuli and sensitive to visual geometry and a longer latency, vection-enhanced postural mechanism, that relies on the conscious perception of self-motion.

Neuroimaging [25] provides further evidence for functional interactions between modality specific brain areas in the perception of self-motion and the generation of postural responses. This is consistent with findings that show how a range of senses, all relying on cortical integration, contribute to postural stability. Stable reference points, whether visual [11, 17, 26], auditory [27, 28] and tactile [29] have been shown to reduce postural sway, we therefore refer to them as ‘postural anchors’.

### Identifying self-motion

To maintain stable stance it is necessary to estimate our own motion relative to the environment. A critical aspect of this self-motion estimation process is the separation of self-motion signals, which must be counteracted, from other, externally generated, motion signals in the environment.

This process is not always successful; a commonly experienced example of illusory motion (vection) is the ‘train illusion’ described by James in 1918 [30]: *“when another train comes alongside of ours in a station*, *and fills the entire window*, *and*, *after standing still awhile*, *begins to glide away*, *we judge that it is* our *train which is moving*.*”* James also observed that the vection illusion strongly depends on environmental and cognitive factors: *“If*, *however*, *we catch a glimpse of any part of the station through the windows*, *or between the cars*, *of the other train*, *the illusion of our own movement instantly disappears”*. An important aspect of postural stability, therefore appears to be the identification of suitable perceptual reference points that provide the ground truth necessary to separate self-motion from externally generated motion signals.

Illusory self-motion affects the overall experience and effectiveness of VR and other immersive environments [31]. Vection can have adverse effects on user experience and may trigger motion sickness, which is typically preceded by postural instability [32].

The presence of appropriate postural anchors that minimise vection in VR may ameliorate the negative effects of vection, therefore improve user experience and performance in simulated environments.

It is an open question how suitable anchors in the environment are chosen to stabilise posture, in particular, whether high level cognitive assessment of the properties of potential anchors contributes to this choice. It is easy to make the case that moving objects, unless motion is highly predictable, are not suitable as postural anchors. Less obvious is the question whether *potentially* moving objects should also be discounted. In this sense any rendered object in a VR environment has more *potential* to move than static real objects, whether this is by design, due to rendering inaccuracies, or because of poor observer tracking.

If an explicit assessment of the suitability of objects as postural anchors is carried out, then the fidelity of rendering is likely to be critical in providing potential anchors to stabilise posture. Objects that are clearly identifiable as ‘unreal’, therefore potentially unstable, should be rejected as potential postural anchors.

While there is significant progress in terms of rendering quality in VR, there are fundamental limitations that are shared by all systems and cannot be overcome. One such limitation is that 3D depth cues are created by providing disparate images to each eye, which can lead to conflicting accommodation and vergence cues when objects are rendered to appear away from the projection plane.

### Disparity cue presentation: Accommodation vs. vergence

**Fig 1:**
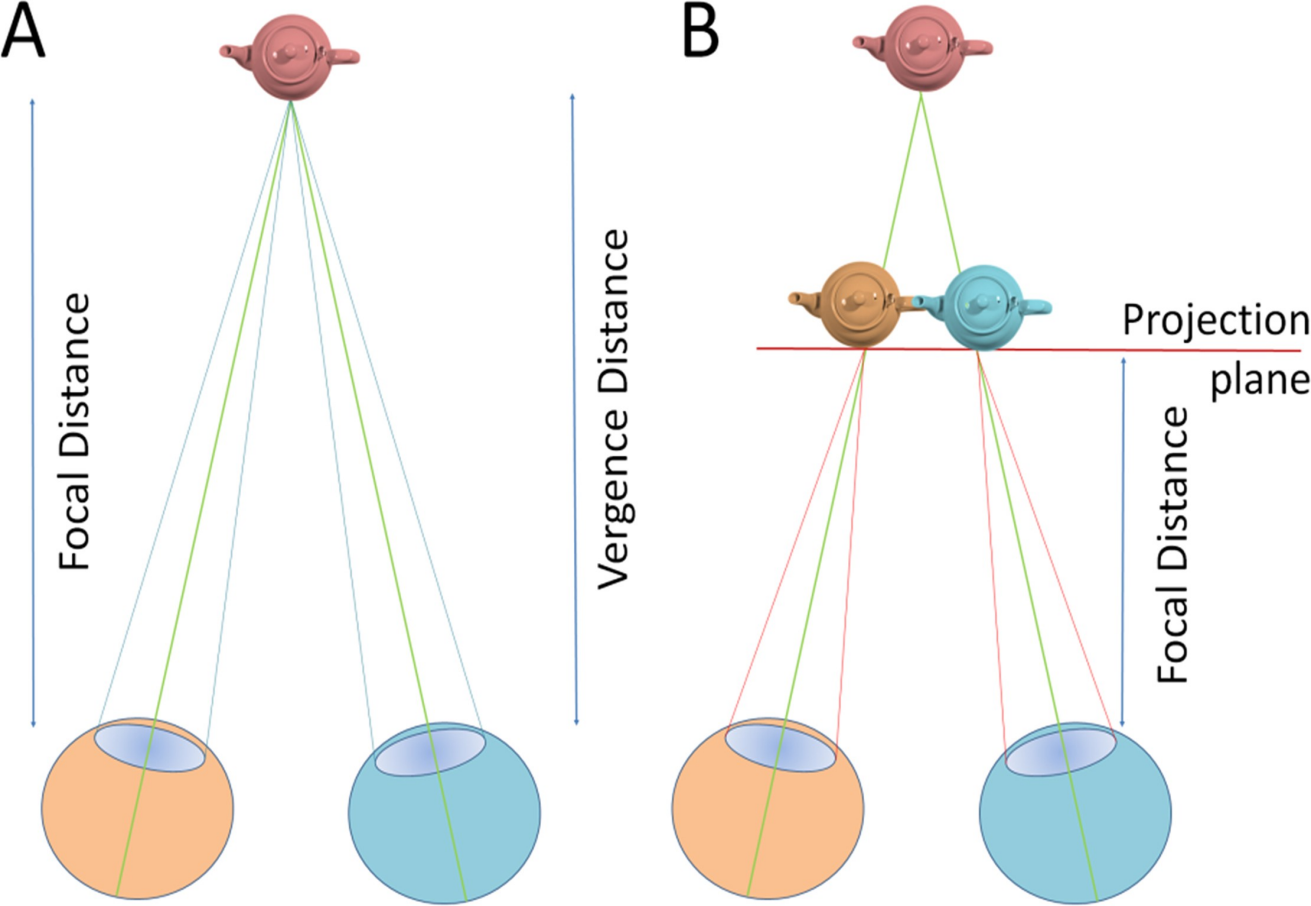
In natural environments accommodation (determined by focal distance) and vergence are linked (a). In most VR environments depth cues are generated by providing disparity cues such that objects are rendered separately for each eye. Accommodation stays on the projection plane, so that conflicting cues are presented which affect performance and comfort.

The perception of depth in natural and virtual environments differs in a number of points. In the real world, in order to see objects clearly, our eyes accommodate (focus) and converge (vergence) onto the target, for real objects the control signals for vergence and accommodation are closely linked [33, 34], Fig 1A.

Rendering visual stimuli in stereoscopic displays is quite different. Most VR environments provide stereoscopic visual cues on a single plane, the projection screen or head mounted display. The illusion of depth is achieved by introducing disparities in the retinal images of the two eyes that result in depth-appropriate vergence eye movements (Fig 1B). While this procedure provides appropriate disparity cues, it cannot provide the necessary accommodation cues because, to see a sharp image, we still accommodate to focus on the display plane. In normal viewing accommodation and vergence are linked while in 3D projection systems conflicting vergence and accommodation control is required for objects that are displaced from the display plane. While viewers are not consciously aware of this accommodation-vergence discrepancy, it is well known that conflicting accommodation-vergence cues cause discomfort and fatigue as well as decreases in performance [35–37].

If mismatching accommodation-vergence cues cause the negative perceptual effects reviewed above, they may also provide the necessary information that identifies as object as unreal and therefore unsuitable for use as a perceptual anchor. The first experiment addresses this question.

### Simulation fidelity and postural stability

A second potential problem that precludes potential postural anchors in VR to be used to stabilise stance is simulation fidelity. Riecke et al. [19] and Blümle et al. [18] showed that postural sway depends on how ‘believable’ visual stimuli in VR are. This is consistent with the explanation that the illusory character of the VR environment affects vection perception and postural responses.

Kapoula et al. [38] on the other hand, compared natural postural sway while observers examined paintings with strong perspective and sense of a recessed space compared to ‘cubist’ renderings, which neutralise rendered depth cues, of the same images and found significantly more postural sway while obervers looked at the original images.

Clear evidence that cognitive processes influence postural control is provided by the finding that *expectation* modulates visually evoked responses, for example when the subject is aware that visual signals causing vection represent external agency rather than by self-motion. Expectation, in contrast does not modulate postural responses driven by the vestibular system. This suggests that afferents provided by the different sensory channels involved in postural control are not similarly susceptible to high level processes such as expectation [20, 26].

### Measuring visually evoked postural responses

Visually evoked postural responses (VEPR) can be measured where perceived self-motion (vection) is counteracted by postural adjustments to maintain stable stance. Since VR systems routinely capture high precision positional information, VEPR provide a convenient and objective measure of behaviour and subjective constructs such as presence or immersion in VR [27].

**Fig 2:**
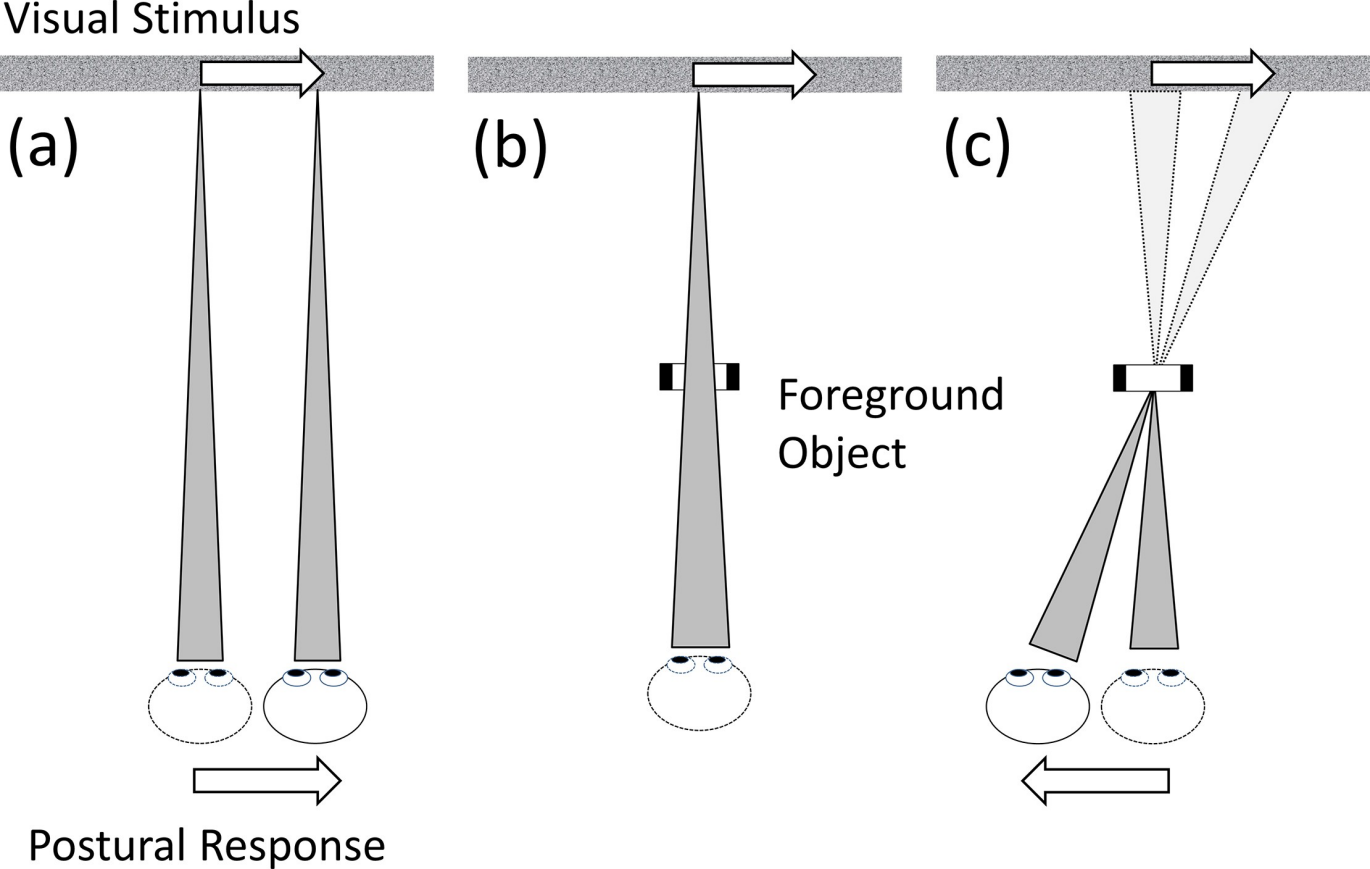
Schematic diagram of the experimental findings that form the basis of our experiments. When participants are fixating a moving background (a) they maintain a constant position relative to it. If a foreground object is present, but not fixated (b), no VEPR are induced. Changing fixation to the static foreground object, however, changes VEPR into the opposite direction as observed in (c).

Our experimental design follows the procedures reported by Bronstein and Buckwell [17] who first provided direct evidence for active, environment-dependent modulation of VEPR. Subjects viewed a laterally moving visual background in three different conditions: (a) direct fixation of the background, (b) fixation of the background through a fixed stationary object (a window) in the foreground, and (c) fixation of the object while the background moved cyclically in the lateral plane (Fig 2).

When only the background was present, participants’ VEPR followed the visual motion signal, as would be expected for visually induced corrective motion that stabilises the observer position relative to an external reference. When participants saw the visual background movement through a fixed object in the foreground, no systematic postural adjustments in response to background movement were observed, as if the static foreground object served as a postural anchor in preference to the moving background. The behavior changed again when participants were asked to fixate the stable foreground object while the background moved (c): in this condition participants moved in the opposite direction as in condition (a), again maintaining a stable relative position between foreground object and the background. Bronstein and Buckwell [17] used these findings as a basis for the argument that control of postural sway does not result from rigid optokinetic reflexes but is modulated by the configuration (and interpretation) of the environment.

### Summary of experimental aims

In a first experiment we explore whether the conflicting vergence and accommodation cues modulate VEPR. The two objects in the simulation, the foreground object and the moving background, were rendered such that in each of two experimental setups one object was projected onto the screen while the other object was either 2m in front or 2m behind the screen, leading to accommodation-vergence conflicts, Fig 2. We hypothesise that fixating the plane with accommodation-vergence conflict identifies visual references as less reliable and therefore reduces VEPR.

In the second experiment we investigate whether an explicit evaluation of the positional stability of the reference points present in the scene modulates VEPR during visual motion stimulation. VEPR in the presence of a potentially unstable helium balloon as a postural reference are compared with those when our standard positional reference is present. We hypothesise that fixating the potentially unstable helium balloon in the foreground should modulate VEPR relative to the stable foreground object.

## Methods

### Ethics statement

The experiments have been approved by the University of Liverpool ethics committee (reference PSYC-1112–049-amended). Written informed consent was acquired from all participants.

### Participants

A total of 24 participants took part in the experiments.

The target conditions tested in experiment 1 are similar to two conditions in previously reported experiments (exp 1a, condition ‘BG visual’, effect size dz = 1.59 and exp 2a, condition ‘BG virtual’, effect size dz = 1.61, [27]). A power analysis to determine the required sample size (G*Power 3.0, [39]), assuming an effect size of dz = 1.6 and an error probability of α = 0.05, shows that eight participants are sufficient to reach an actual power 1-β = 0.97. Eleven participants, aged from 18 to 52, (M = 28.7, SD = 13.24; 7 males), were recruited.

Comparable experimental data was not available for the conditions tested in experiment 2, to allow for a reduction in effect size by 30% to dz = 1.1, 13 participants, aged from 20 to 58, (M = 29.9, SD = 15.45, 9 males), were recruited.

All participants were recruited using opportunity sampling and all signed a consent form. All participants reported normal or corrected-to-normal vision. No participant took part in both experiments. The participants were not aware of the experimental hypothesis.

### Virtual reality set up

The laboratory consisted of a planar display screen of 6.0m wide and 2.1m high with two active stereo back-projectors that displayed 3390 x 1200 resolution images at a rate of 120Hz. 3D stereo images were rendered by an NVIDIA Quadro K6000 GPU. Observers wore wireless LCD shutter glasses that were synchronized with the projectors to provide stereoscopic images. The position of the shutter glasses was tracked using 16 VICON Bonita B3 infrared cameras (250 fps capture speed, motion resolution of 0.5mm of translation and 0.5 degrees of rotation in a 6m x 6m volume using 9mm markers). Position data, computed using VICON Tracker software, was broadcast in real-time across the internal network using a VRPN protocol at a rate of 200Hz and used to update the virtual environment. The position of the 3D-glasses in space was recorded to provide the head position data used in the analysis of postural sway.

**Fig 3:**
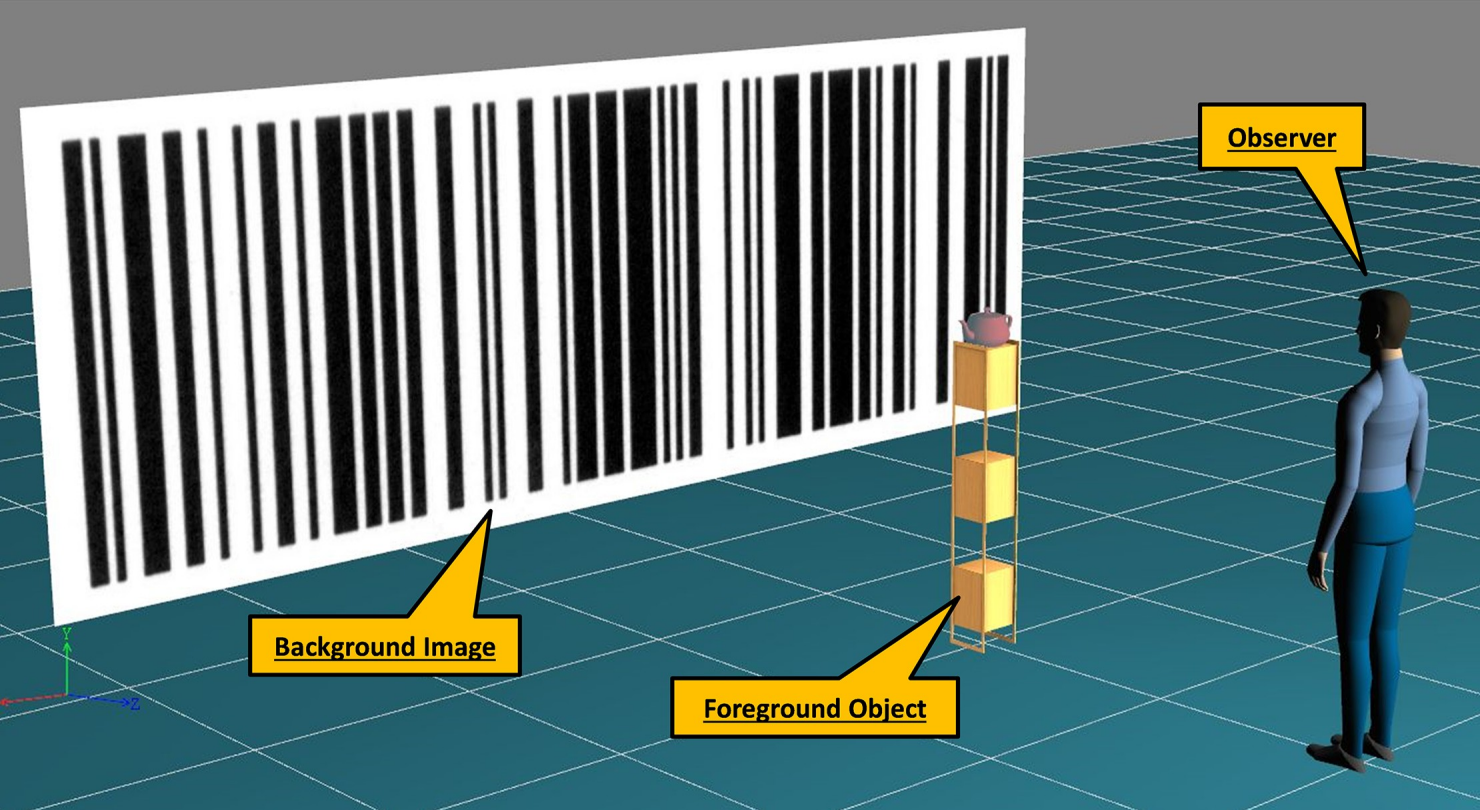
Elements of the virtual reality environment used in all experiments: The translating background image (a barcode), the foreground object (a virtual or real teapot on a stand) and an avatar representing the observer in the scene.

The virtual reality environment (Fig 3) consists of three major elements:

*The background image*: an image of a barcode extending 6m x 2.1m (86° horizontal visual angle). The high contrast barcode image was chosen to give strong lateral motion cues.

*The foreground object*: a real object or a 3D geometric model of a teapot on a stand, 2m from the observer subtending 4.6° visual angle (Fig 3). Real and virtual objects were matched in size and position. One of the real foreground images used in experiment 2 was helium balloon that appeared to be free floating, but was, in reality, fixed in position from behind. Participants had to pass a number of free floating matching balloons on their way to the experimental position to emphasise that the foreground object had the potential to move. The free floating balloons were behind the observer and therefore not visible during the experiment.

*The observer*: a virtual camera whose position and orientation are directly linked to that of the real life observer head position through the tracking system to dynamically render images on the display screen that will appear to the observer as a true perspective of the scene. The avatar shown in Fig 3 is not part of the experimental setup. The relative positions, but not the position of the projection plane, was the same as in [27]; Motion tracking and rendering hardware were significantly improved.

### Stimuli

Lateral movement of a background image causes highly environment-dependent VEPR [17, 27]

In this study, the virtual environment was manipulated in two experiments each consisting of two experimental blocks containing medio-lateral visual motion of the background image. The order of presentation was counterbalanced across the participant pool. Each block lasted 20 minutes and participants had a minimum of 20 minutes rest between the two successive blocks. Within each block, five conditions were run that consisted of 20 presentations of a 6 second visual motion pattern that preceded 6 seconds of a static display (rest condition) during which natural postural sway was measured. The dynamic characteristics of the visual stimulus follows previous studies and was chosen to be within the range of spontaneous body sway, consistent with the aim to present motion signals that would be interpreted as being caused by self-motion. The motion signals consisted of three raised-cosine oscillations at a frequency of 0.5Hz and maximum amplitude of 50mm (0.7° visual angle) relative to the origin [27].

All experiments used the same motion signals. The only differences between the experimental blocks were the vergence plane (foreground or background object, experiment 1) or the implied stability of the real foreground object (teapot vs balloon, experiment 2).

### Procedure

**Fig 4:**
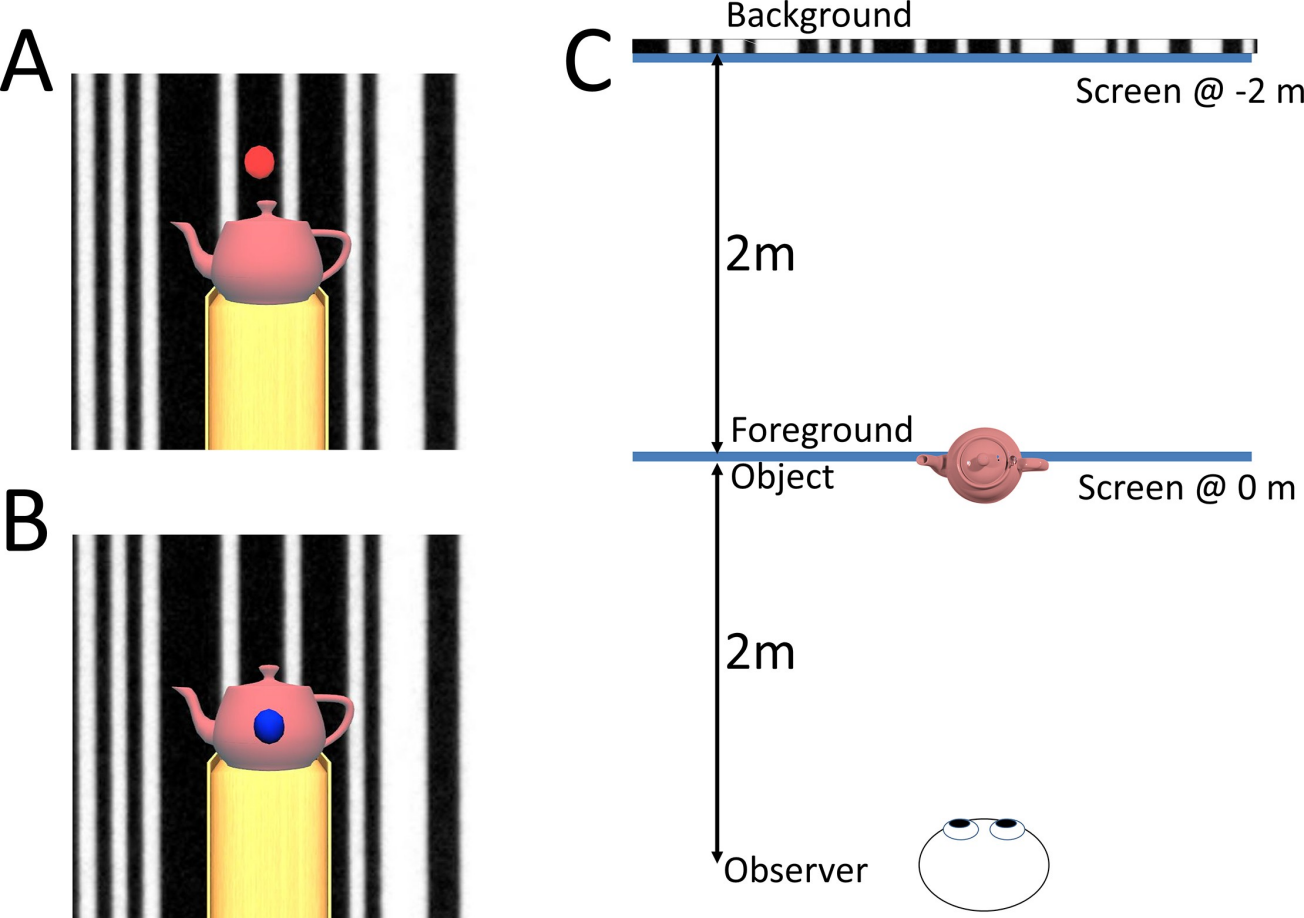
The stimuli from the viewer’s perspective. Participants were asked to press a button when the fixation target (either a red dot on the background image (A) or a blue dot on the foreground object (B)) transiently disappeared. Panel C shows a plan view of the room layout: Participants viewed a moving image of a barcode 4m ahead of them. In contrast to a window that participants had to look through [17], a real or virtual object 2m from the observer was used as a foreground object. In experiment 1 we tested two conditions: in one condition the projection screen was situated 2.0 metres in front of the participants, such that the foreground object had matching accommodation and vergence cues while in a second condition the projection screen was 4.0 m from the observer such that the background was ‘correctly’ displayed.

Participants wore LCD shutter glasses and were asked to stand on a foam pad with both feet approximately 20cm apart. Foam padding was used to enhance VEPR [40]. Participants were asked to keep as still as possible and fixate on the target. The distance from the projection screen was varied: during the 0m experimental block they were standing 4m from the projection screen; during the -2m experimental block they were standing 4m from the projection screen (Fig 4). Streepey et al. [41] previously reported that peripheral vision plays an important role in postural stabilisation. To control for this, the field of view was restricted by “blinkers” attached to the 3D shutter glasses limiting the field of view to approximately 75 degrees in experiment 2.

Participants were asked to fixate a target (either a red or blue dot), and press a button when the fixation target transiently disappeared, to ensure that they maintained focus on the appropriate area of the virtual display. The disappearance of the fixation point was elicited at random intervals in all conditions during moving and static displays. There was approximately 1 event every 5 seconds and with at least 1 second minimum gap between events.

### VEPR analysis

Head position data was recorded using a VICON motion tracking system using IR reflective markers on the 3D shutter glasses worn by the participants. To quantify the VEPR from the recorded postural sway data, natural sway components have to be accounted for. Modulation by lower-limb proprioceptive signals leads to very low frequency sway (<1Hz) and 95% of the energy in natural sway is at a frequency less than 2Hz [42]. To select for induced sway responses, the recorded data, sampled at 10Hz, was filtered off-line using a second order zero-lag Butterworth band-pass filter with cut off frequencies of 0.125Hz (LF) and 2.5Hz (HF).

To quantify the VEPR, the proportion of motion energy at the visual stimulus frequency (0.5Hz) relative to the total energy in the spectrum was computed (eqn. 1) for the stimulus and rest conditions. The analysis windows in both cases extended over the full 6 seconds of stimulus or rest.

$$VEPR{}_{v}=\frac{F(v)}{\sum_{w=0}^{Fs/2}F(w)}$$

Where *VEPR* is the amplitude of the Fourier component, *F(x)*, at the visual stimulus frequency, *v = 0*.*5Hz*, relative to the total energy in the spectrum between 0 Hz and half the sampling frequency (*F*_*s*_*/2 = 5Hz*).

The motion stimulus followed a raised cosine trajectory, such that the maximum deflection was seen after 1 sec. The phase of the Fourier component, ω(x), at the visual stimulus frequency consequently provides a measure of whether participants move with the moving background or in antiphase as reported for experimental conditions where participants fixate on a stable foreground object [17].

The motion signal, recorded by the VR motion tracker, contains stimulus evoked postural responses, but also a significant amount of spontaneous sway. To isolate the stimulus driven motion component, motion signals were averaged over 20 epochs, each of 6 sec duration for each stimulus condition. The data were filtered using zero phase delay, second order Butterworth filter with a passband between 0.25 Hz and 5 Hz. To exclude large spontaneous motion signals, such as when participants changed posture, any epochs with a total motion signals greater than 2 sd of the participant average were excluded. The analysis described in more detail in [27].

### Statistical analysis

Both experiments shared common designs and analysis.

The analysis for both experiments was conducted in two stages: In a first stage an ANOVA is used to identify whether there are significant main effects of visual motion presentation in the lateral direction. Four factors were used in each case: ‘experimental condition’ (2) x ‘environmental configuration (5) x ‘visual stimulus motion’ (2) and ‘subject’ as a random factor. Analysis details are given in tables A (exp 1) and E (exp 2) in S1 File

Planned comparisons for specific conditions were then carried out where significant main effects or interactions were found. Full details agre given in tables B-D (exp 1) and F-H (exp 2) in S1 File.

To isolate the visually evoked motion from random motion, all motion signals were time aligned with the onset of the visual stimulus motion and averaged. This means that visually evoked components will add, while random, non-visually evoked motion will average to zero. The VEPRs statistics were computed using the population response. Bootstrapping was used to estimate the sample population mean and variance of the VEPR [43]. All statistical tests on these population estimates were performed using independent (two-sample) t-tests [44]. Appropriate Bonferroni correction was used in these tests.

## Results

### Experiment 1. Accommodation-vergence conflict modulates postural sway

The behavioural performance of participants was recorded as the percentage of correct responses identifying when the fixation point transiently disappeared. Overall, participants correctly identified more than 98% of the events (M = 98.15, SD = 1.55). This means that attention or fixation position as a cofounding variable can be rejected.

Two experimental conditions were run in quasi-random sequence. In one condition the moving background was at the projection screen (0m), causing an accommodation-vergence conflict only for the virtual foreground object; in the other condition (-2m) the moving background as 2m behind the projection screen while the foreground object was projected at the screen, Fig 4.

Within each block, five environment configurations (BG only, BG virtual, BG real, FG virtual, FG real) were tested. They differed in terms of the fixation point position (BG: fixation point on the moving background, FG: fixation point on the foreground object) and the nature of the foreground object which could be none (BG only), virtual or real.

A repeated-measures analysis of variance (ANOVA) was conducted with the factors ‘vergence’ (-2m or 0m), ‘visual motion’ (on or off), ‘condition’ (the five fixation conditions) and ‘subject’ as a random factor. The analysis showed significant main effects of vergence (F(1,159) = 4.86, p = 0.0291), visual motion (F (1,159) = 21.13, p < 0.0001) and condition (F (4,159) = 2.50, p = 0.045). Details of the ANOVA are shown in the supporting information (S1 File).

Ten planned comparisons, with Bonferroni correction, where conducted to quantify the VEPR component at 0.5Hz in comparison to the rest condition (pause). One-sided t-tests were used because we expect VEPR to be larger in the motion condition than during rest.

**Fig 5:**
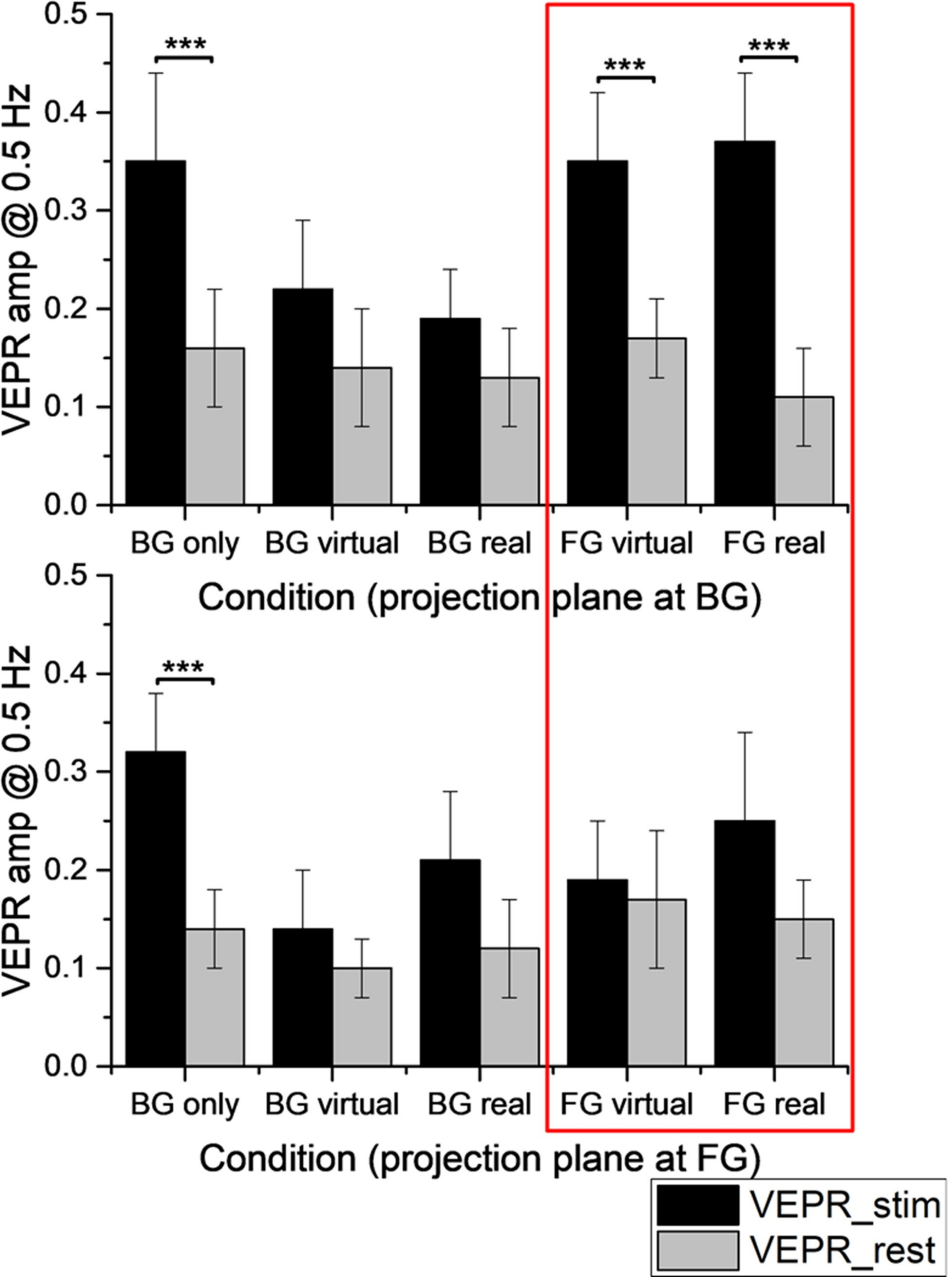
Relative amplitude of postural responses to visual stimulus in motion (black bars) and in pause (grey bars) in the two experimental blocks. The top graph shows the VEPR component at 0.Hz relative to rest (grey) in the five conditions when the background image was on the projection plane. The lower graph shows equivalent data for the block where the fixation plane was at the position of the foreground object. When the background is coincident with the projection plane, significant VEPR are seen when no foreground is present or when the foreground is fixated. No significant VEPR are seen when participants fixate the foreground (on the fixation plane) when the background is 2m behind the projection screen (red rectangle).

For the 0m condition (top line of Fig 5), where the moving background was on the projection screen, the observed data matches the findings first described by Bronstein and Buckwell [17]: significant VEPR (*** for p < 0.001 in Fig 5, top) are elicited in the ‘BG only’ condition and when participants fixate a foreground object (FG real and FG virtual). No significant VEPR are seen when participants fixate the background in the presence of a stable foreground object (BG real and BG virtual). These findings extend the original findings [17] by showing matched behaviour for real and virtual foreground objects. Tables showing the raw data, and detailed results of the statistical analysis for all conditions can be found in the supporting information (S1 File).

Changing the environment configuration, such that the foreground object is on the projection screen while the moving background image is presented 2 meters behind the screen (-2m condition), changes VEPR patterns recorded for the same participants (lower panel, Fig 5).

The main difference between the two experimental conditions is that significant VEPR are seen in the ‘FG real’ and ‘FG virtual’ conditions when the moving background is presented at the display plane (0m condition), but no significant VEPR are seen when the moving background is project 2m behind the display plane. A direct comparison across the two experimental conditions shows significantly higher VEPR amplitudes in the ‘FG virtual’ (t_20_ = 2.6915, p = 0.014) and ‘FG real’ (t_14_ = 4.66, p < 0.001). We conclude that conflicting accommodation-vergence cues for the moving background significantly reduce VEPR amplitude.

VEPR when participants fixate on the background, with or without postural reference signals in the foreground, are not modulated by the position of the display plane. Detailed statistics are shown in table D of S1 File. This suggests that it is not the accommodation-vergence conflict that modulates VEPR, but instead an assessment of the relative positional stability of objects in the environment.

**Fig 6:**
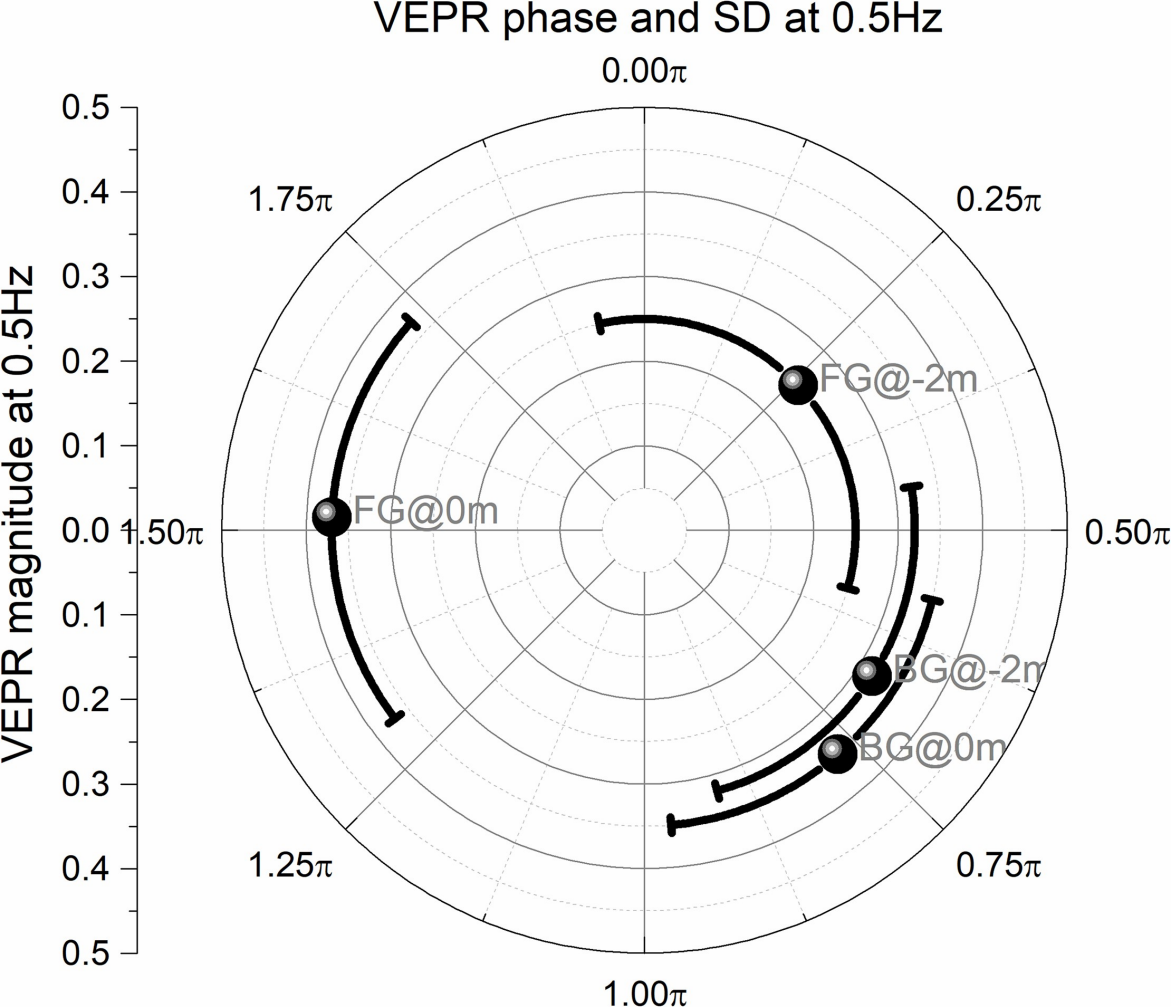
Sway amplitude and phase (error bars SD of phase) in the two experimental conditions. When only the moving background is present (BG @0m and BG @-2m) VEPR amplitude and phase are similar. When participants fixate a stable foreground object and the moving background is on the projection screen (FG @0m) the evoked VEPR are significantly different and almost in antiphase (-0.54*π* vs -+0.58*π*) to the BG condition. This is not the case when the moving signals is placed 2m behind the object and projection screen (FG @-2m).

An important observation in the original experiments [17] was that the phase of sway changed between conditions where the background alone and where a stable (real) foreground object was fixated. Fig 6 shows the VEPR phase and amplitude (error bars SD of phase estimates) for the four conditions where a virtual foreground object was present. The VEPR phase is similar ω(0.5) = 0.58*π* (BG@0m) ω(0.5) = 0.70*π* (BG@-2m) and when no foreground object is present and participants fixate the background. This delay in the response relative to the stimulus is consistent with data reported by Bronstein and Buckwell [17]. When a virtual foreground object is present and the background is at the projection screen, VEPR amplitude is similar (F(0.5) = 0.35) to that seen when no foreground object is present (F(0.5) = 0.35), but the VEPR phase changes to ω(0.5) = 1.46*π* or -0.54*π* (FG@0m, Fig 6). This means that participants move in almost the opposite direction when fixating on the foreground object compared to VEPR when only the moving background is present. When the moving background is projected at -2m, the sway amplitude is significantly lower and not significantly different from the natural sway when no foreground object is present. The mean phase is ω(0.5) = 0.42*π*.

### Experiment 2. Stability assessment modulates VEPR

The aim of Experiment 2 was to investigate whether the perceived stability of real environmental anchors presented as foreground objects modulate VEPR. The key experimental condition therefore is a comparison of the effect of the real (stable) teapot on a stand with VEPR elicited when a potentially unstable helium balloon was present as a foreground object while participants fixate the background.

As in experiment 1, participants were asked to respond to a transiently disappearing fixation target to ensure they were attending the stimuli. All participants correctly identified 99.57% of events (SD = 6.13) from which 99.48% (SD = 6.89) in the teapot block and 99.65% (SD = 5.42) in the balloon block. This means that the attention or fixation position as a confounding variable can be rejected.

A repeated measures ANOVA with factors ‘foreground stability’ (stable, unstable), ‘viewing condition’ (BG only, FG real, FG virtual, BG real, BG virtual) and ‘visual stimulus’ (ON, OFF) and ‘subject’ as a random factor showed a significant main effect of visual stimulus (F(1, 319) = 17.53, p < 0.0001). There were no significant interactions. Full ANOVA tables for the experiment are given in the supporting information, S1 File)

**Fig 7:**
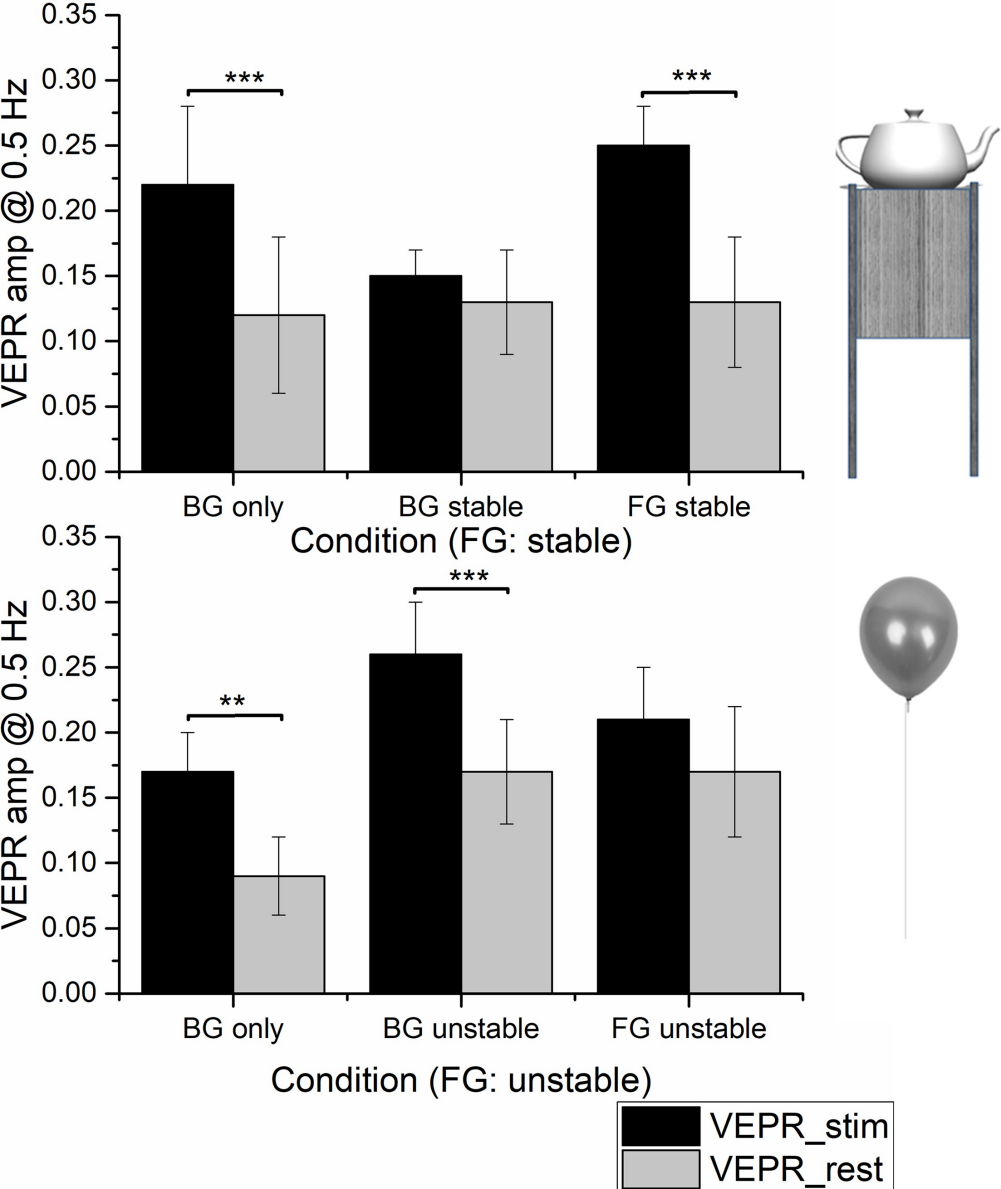
Relative VEPR amplitude (black bars) and spontaneous motion in the ‘rest’ condition (grey bars). In both conditions significant VEPR are elicited when only the background is present. When a stable real foreground object (a teapot on a stand) is present, significant (antiphase) VEPR are evoked when participants fixated the FG object but no systematic VEPR are seen when participants attend the background. The opposite pattern is seen when a (static) balloon is presented as a FG object. The labels identify whether the fixation was on foreground or background.

Fig 7 shows the recorded VEPR in the background only (BG only) and two condition where real objects (‘FG real’ and ‘BG real’) for experimental conditions where either the stable teapot (top line, Fig 7) or the potentially unstable helium balloon (lower line, Fig 7) served as the foreground object.

When the teapot was present as a stable foreground object, participants behaved as in experiment 1 and in the original study [17]. Significant VEPR (t_(24)_ = 4.25, p = 0.0003) are seen in the ‘BG only’ condition, no significant VEPR are recorded when a stable foreground object is present and participants fixate the background immediately behind the foreground object. When fixation changes to the foreground object, significant sway (t_(24)_ = 5.05, p = 0.0001), in the opposite phase as for the ‘BG only’ condition, is seen. When the stable reference is replaced with a helium balloon, the relative VEPR magnitude changes, Fig 7. In this condition significant VEPR are seen when participants fixate the background immediately behind the foreground object (t_(24)_ = 5.73, p = 0.0001), while in the condition where participants fixate the foreground significant VEPR are no longer recorded. Full statistical tables are given as tables F-H in S1 File.

A direct comparison of the visually evoked sway for the two types of foreground objects shows significantly more sway (t_(14)_ = 6.96,p< 0.0001) when the background was fixated immediately behind the balloon and significantly more sway when fixation changed to the stable foreground object (t_(24)_ = 2.47, p = 0.021).

Overall the results are consistent with the hypothesis that observers explicitly evaluate the potential stability of objects in the environment and adapt postural control strategies accordingly.

## Discussion

The primary aim of this study was to investigate how explicit cognitive appraisal of the configuration of real and virtual environments modulates automatic visually evoked postural responses.

The principal finding of experiment 1 is that in 3D virtual environments, where accommodation-vergence conflicts are commonly observed, the location of visual stimulus evoking postural responses relative to the projection plane significantly modulates postural responses.

Kapoula et al. [38] investigated the effects of pictorial depth in static 2D pictures on postural stability and showed greater natural body sway when participants watched pictures that represented ‘depth’ than for control images where depth cues were neutralized. They also report that body sway was larger when participants fixated the recessed part of a painting than when they looked at the foreground area. In the context of our data, a possible explanation for this finding is that pictorial representations of distant scenes are less effective at providing ‘postural anchors’. These areas, represented on 2D pictures, of course, also contain significant accommodation-vergence conflict. The failure to stabilise posture is analogous to the failure of our moving backgrounds to evoke postural responses.

The effect we observe is asymmetric, an accommodation-vergence conflict of the background ‘wallpaper’ modulates VEPR when participants look at the foreground object, whether it is real or virtual. When participants look at the foreground while the background moves, no significant postural responses are seen—it makes no difference whether the foreground object is real or virtual and whether the foreground or background object are presented with conflicting accommodation-vergence cues.

This finding suggest that accommodation-vergence conflict suppresses normally occurring postural responses because the introduction of accommodation-vergence conflict does not introduce additional VEPR in situations where the presence of a foreground object suppresses systematic VEPR.

Matheron et al. [45] showed that even small vertical disparity has a notable influence on postural stability compared to normal eye alignment, and attribute the effect to sensory processing of disparity and/or to oculomotor signals triggered by the disparity. This is consistent with the finding that, in addition to retinal slip, gaze position and convergence are involved in postural stabilization [46] at relatively short distances. All objects used in our experiments are outside the range where vergence cues have been shown to affect postural stability.

An *implicit* assessment of the plausibility of the motion signal, via the detection of accommodation-vergence conflict, therefore appears to be a better explanation for the modulation of VEPR.

An explicit assessment of the stability of potential reference objects as postural anchor was explored in experiment 2. VEPR were recorded in matching environments for two real and positionally stable foreground objects. One, a teapot on a stand, was designed to provide an obvious stable reference, the other, a helium balloon, was designed to suggest potential motion. It has previously been shown that fixating a moving background through a stable foreground significantly reduces or abolishes VEPR [17, 24, 27]. For our potentially unstable foreground object, VEPR are comparable to those seen when only the moving background was visible were recorded. When a stable foreground object is fixated during background motion, participants exhibit VEPR that are consistent with behaviour that minimises relative movements of foreground and background object–participants sway in the opposite direction to the motion induced when no foreground is present. No significant VEPR are seen when participants fixate a potentially unstable foreground object.

The behaviour seen in both conditions is consistent with the hypothesis that an explicit assessment of the stability of the foreground object modulates VEPR. An alternative or additional explanation for the differential behaviour is that the nature of the object (volume, shape, complexity) influences VEPR: it could, for example, be argued that the smooth, floating balloon provides fewer useful cues for the control of postural stability than the teapot stimulus. In the experiments reported here participants looked at a laser dot in the centre of the real objects and reported the transient disappearance of this dot. Visual acuity, and presumably the ability to detect small movements, declines with increasing eccentricity, so that the object edges nearest the fixation point, which were similar for both objects, are likely to have a dominant effect. The train illusion [30] shows that we dynamically judge stability of environmental anchors, so the paradigm described here may provide a tool to study in more detail what features determine the perceived stability of environmental objects or their suitability as postural anchors.

A better understanding of the effects modulating postural sway, or, in real environments, features that maximise postural stability may in time contribute to the design of screening tests that identify vulnerable populations, or to the design of guidelines for built environments that, for example, minimise falls in the elderly by maximising visual cues that are available for postural control. Virtual reality systems have already been shown to provide a more enjoyable tool for vestibular system rehabilitation than conventional methods, but the outcomes of both approaches are not significantly different [47, 48]. A thorough theoretical understanding of what factors influence the stability of VR representations and how that can be manipulated may increase be effectiveness of VR rehabilitation beyond that of current alternative therapies.

The data explain previous findings employing the same methodology [27] where significant differences in VEPR were seen for real and virtual objects that were presented in the foreground. The data presented here (experiment 1, Fig 5) shows no difference between the VEPR for real and virtual foreground objects and matching behaviour for both object classes when the projection plane is changed to manipulate accommodation-vergence conflict. While the physical dimensions of the VR systems and virtual environment are the same in this and the previous study, there were significant upgrades to the motion tracking (more cameras and faster communications), the display equipment and rendering software. Finding matching behaviour (and VEPR) for the real and virtual foreground objects reinforce the argument that VEPR are an excellent way to measure presence and immersion in virtual environments: The measurements rely on precise participant tracking, which is a component in any high quality VR system, in response to well defined visual motion signals, these can very easily be manipulated by systematically changing the ‘camera’ position in VR simulations. The experiments quantify objective behaviour, visual evoked postural sway, that participants are not overtly aware of, they therefore complement other subjective evaluations, such as presence or immersion questionnaires [49, 50].

### Implications for the design of virtual environments

It has previously been reported [18, 19, 27] that visually evoked postural responses in virtual environment differ from those seen in real environments. Head position data is routinely tracked with high precision in VR systems, so that VEPR provide a convenient measure of simulation fidelity, presence and immersion [27].

We identify two factors that modulate VEPR in VR environments:

Data from experiment 1 show ‘natural’ VEPR only when the moving background signals are displayed on the projection plane. This suggests that motion signals exhibiting accommodation-vergence conflict are less effective in evoking postural responses. Appropriate placement of motion signals in the VR scene can therefore be used to either enhance or minimise vection. It has previously been argued that VEPR are a convenient measure for presence in VR environments [27]. Appropriate placement of signals, intended to cause vection, therefore may modulate presence in mediated environments.

Experiment 2 shows that we explicitly assess the stability of objects that potentially serve as reference points for postural control. While there have been considerable improvements in rendering in recent years, there is no question that in most environments virtual, and consequently potentially unstable, objects can easily be recognised as such. Virtual and real foreground objects cause similar VEPR in experiment 1, which shows that objects, even if they are clearly recognisable as virtual or rendered objects, are used as postural anchors. We hypothesize that observed object motion (as seen in the free floating balloons in our experiment 2, or the moving train in James’ illusion) is what identifies objects as being unsuitable as postural anchors. This is good news for VR design, because it means that surface fidelity is not a key requirement to produce realistic VEPR. If the detection of object motion, however, excludes objects from being a potential postural anchor, then this places strict design requirements on motion tracking and rendering subsystems in VR.

Inaccurate motion tracking or sluggish rendering will cause signals that can be identified as object motion; high precision tracking and rendering is therefore a key requirement. Participants in our experiments were exposed to freely moving balloons when they entered the VR lab while the balloon that was used as a foreground reference was held rigidly in position without the participant’s knowledge. A sensible heuristic would be to assume that inanimate objects are generally stationary (for example railway carriages) *until* motion is detected and to assume that once an object is detected as moving, it should be excluded from a potential postural anchor for some period. This would explain why the train illusion persists and why participants ignored the balloon in experiment 2.

In many VR systems accurate motion tracking is limited to a specific space, and typically relies on markers attached to the head mounted display or 3D glasses. If, as experiment 2 suggests, persistent decisions about object stability that affect their use as postural anchors, are made, then it is important that all objects in VR simulations are stable at all times. A critical point is the start of a simulation where observers typically put (tracked) shutter glasses on or move into the space where motion capture is precise. Inaccuracies in tracking or motion signals caused when, for example, tracked shutter glasses cause inappropriate movement signals may have a lasting deleterious effect on user experience in VR. Simulations should therefore only provide signals when markers used for observer tracking are firmly in place and the observer is in a space that can be tracked with high accuracy.

## Conclusion

The experimental framework for evaluation of fidelity in VR that includes the assessment of postural responses has been proposed previously [27]. Our findings support this notion and further confirm that postural responses can be affected by implicit and explicit assessment of object behaviour in real and virtual environments. In particular, we showed that visual disparity cues that are present in virtual environments are important factor in modulating VEPR. Our data support the hypothesis that viewers make explicit and lasting assessments whether objects in the environment provide the necessary positional stability to serve as postural anchors. The presentation of visual information should therefore be considered carefully in order to avoid adverse effects of self-motion.

## Supporting information

S1 File Detailed statistical analysis for both experiments. (DOCX)

## References

[1] Kavounoudias A, Roll R, Roll JP. Foot sole and ankle muscle inputs contribute jointly to human erect posture regulation. The Journal of physiology. 2001;532(3):869–78. 11313452 10.1111/j.1469-7793.2001.0869e.x PMC2278585

[2] Allum J, Bloem B, Carpenter M, Hulliger M, Hadders-Algra M. Proprioceptive control of posture: a review of new concepts. Gait & posture. 1998;8(3):214–42. 10200410 10.1016/s0966-6362(98)00027-7

[3] Hassall CD, MacLean S, Krigolson OE. Hierarchical error evaluation: the role of medial-frontal cortex in postural control. Journal of motor behavior. 2014;46(6):381–7. 10.1080/00222895.2014.918021 25205434

[4] Jacobs J, Horak F. Cortical control of postural responses. Journal of neural transmission. 2007;114(10):1339–48. 10.1007/s00702-007-0657-0 17393068 PMC4382099

[5] Dichgans J, Brandt T. Visual-vestibular interaction: Effects on self-motion perception and postural control Perception: Springer; 1978 p. 755–804.

[6] Dijkstra T, Schöner G, Giese MA, Gielen C. Frequency dependence of the action-perception cycle for postural control in a moving visual environment: relative phase dynamics. Biological Cybernetics. 1994;71(6):489–501. 7999875 10.1007/BF00198467

[7] Bacsi AM, Colebatch JG. Evidence for reflex and perceptual vestibular contributions to postural control. Experimental brain research. 2005;160(1):22–8. 10.1007/s00221-004-1982-2 15322784

[8] Cenciarini M, Peterka RJ. Stimulus-dependent changes in the vestibular contribution to human postural control. Journal of neurophysiology. 2006;95(5):2733–50. 10.1152/jn.00856.2004 16467429

[9] Mahboobin A, Loughlin PJ, Redfern MS, Sparto PJ. Sensory re-weighting in human postural control during moving-scene perturbations. Experimental Brain Research. 2005;167(2):260–7. 10.1007/s00221-005-0053-7 16025292

[10] Peterka R. Sensorimotor integration in human postural control. Journal of neurophysiology. 2002;88(3):1097–118. 10.1152/jn.2002.88.3.1097 12205132

[11] Howard IP, Howard A. Vection: the contributions of absolute and relative visual motion. Perception. 1994;23(7):745–51. 10.1068/p230745 7845766

[12] Lestienne F, Soechting J, Berthoz A. Postural readjustments induced by linear motion of visual scenes. Experimental Brain Research. 1977;28(3):363–84. 885185 10.1007/BF00235717

[13] Van Asten W, Gielen C, Van Der Gon JD. Postural adjustments induced by simulated motion of differently structured environments. Experimental Brain Research. 1988;73(2):371–83. 3215313 10.1007/BF00248230

[14] Berthoz A, Lacour M, Soechting J, Vidal P. The role of vision in the control of posture during linear motion. Progress in brain research. 1979;50:197–209. 10.1016/S0079-6123(08)60820-1 551426

[15] Guerraz M, Gianna CC, Burchill PM, Gresty MA, Bronstein AM. Effect of visual surrounding motion on body sway in a three-dimensional environment. Perception & psychophysics. 2001;63(1):47–58. 11304016 10.3758/bf03200502

[16] Day BL, Muller T, Offord J, Di Giulio I. Dual processing of visual rotation for bipedal stance control. The Journal of physiology. 2016;594(19):5661–71. 10.1113/JP271813 27686250 PMC5043039

[17] Bronstein A, Buckwell D. Automatic control of postural sway by visual motion parallax. Experimental Brain Research. 1997;113(2):243–8. 9063710 10.1007/BF02450322

[18] Blümle A, Maurer C, Schweigart G, Mergner T. A cognitive intersensory interaction mechanism in human postural control. Experimental brain research. 2006;173(3):357–63. 10.1007/s00221-006-0384-z 16491407

[19] Riecke BE, Schulte-Pelkum J, Avraamides MN, Heyde MVD, Bülthoff HH. Cognitive factors can influence self-motion perception (vection) in virtual reality. ACM Transactions on Applied Perception (TAP). 2006;3(3):194–216.

[20] Guerraz M, Day BL. Expectation and the vestibular control of balance. Journal of cognitive neuroscience. 2005;17(3):463–9. 10.1162/0898929053279540 15814005

[21] Guerraz M, Thilo KV, Bronstein AM, Gresty MA. Influence of action and expectation on visual control of posture. Cognitive Brain Research. 2001;11(2):259–66. 11275487 10.1016/s0926-6410(00)00080-x

[22] Caudron S, Boy F, Forestier N, Guerraz M. Influence of expectation on postural disturbance evoked by proprioceptive stimulation. Experimental Brain Research. 2008;184(1):53–9. 10.1007/s00221-007-1079-9 17703285

[23] Melzer I, Benjuya N, Kaplanski J. Age-related changes of postural control: effect of cognitive tasks. Gerontology. 2001;47(4):189–94. 10.1159/000052797 11408723

[24] Bardy BG, Warren WH, Kay BA. Motion parallax is used to control postural sway during walking. Experimental Brain Research. 1996;111(2):271–82. 8891657 10.1007/BF00227304

[25] Guerraz M, Bronstein AM. Mechanisms underlying visually induced body sway. Neuroscience letters. 2008;443(1):12–6. 10.1016/j.neulet.2008.07.053 18672020

[26] Slobounov S, Wu T, Hallett M, Shibasaki H, Slobounov E, Newell K. Neural underpinning of postural responses to visual field motion. Biological Psychology. 2006;72(2):188–97. 10.1016/j.biopsycho.2005.10.005 16338048

[27] Meyer GF, Shao F, White MD, Hopkins C, Robotham AJ. Modulation of visually evoked postural responses by contextual visual, haptic and auditory information: a ‘virtual reality check’. PloS one. 2013;8(6):e67651 10.1371/journal.pone.0067651 23840760 PMC3695920

[28] Palm H-G, Strobel J, Achatz G, von Luebken F, Friemert B. The role and interaction of visual and auditory afferents in postural stability. Gait & posture. 2009;30(3):328–33. 19592254 10.1016/j.gaitpost.2009.05.023

[29] Kouzaki M, Masani K. Reduced postural sway during quiet standing by light touch is due to finger tactile feedback but not mechanical support. Experimental brain research. 2008;188(1):153–8. 10.1007/s00221-008-1426-5 18506433

[30] James W. The principles of psychology: Read Books Ltd; 2013.

[31] Bernhard ER. Compelling self-motion through virtual environments without actual self-motion–Using self-motion illusions ('vection') to improve VR user experience 2011.

[32] Villard SJ, Flanagan MB, Albanese GM, Stoffregen TA. Postural instability and motion sickness in a virtual moving room. Human factors. 2008;50(2):332–45. 10.1518/001872008X250728 18516843 PMC4030407

[33] Lambooij M, Fortuin M, Heynderickx I, IJsselsteijn W. Visual discomfort and visual fatigue of stereoscopic displays: A review. Journal of Imaging Science and Technology. 2009;53(3):30201-1—14.

[34] Lambooij M, Fortuin M, Ijsselsteijn W, Evans B, Heynderickx I. Measuring visual fatigue and visual discomfort associated with 3‐D displays. Journal of the Society for Information Display. 2010;18(11):931–43.

[35] Hoffman DM, Girshick AR, Akeley K, Banks MS. Vergence–accommodation conflicts hinder visual performance and cause visual fatigue. Journal of vision. 2008;8(3):33 10.1167/8.3.33 18484839 PMC2879326

[36] Banks MS, Kim J, Shibata T, editors. Insight into vergence/accommodation mismatch Head-and Helmet-Mounted Displays XVIII: Design and Applications; 2013: International Society for Optics and Photonics. 10.1117/12.2019866 PMC3824256 24244832

[37] Shibata T, Kim J, Hoffman DM, Banks MS, editors. Visual discomfort with stereo displays: effects of viewing distance and direction of vergence-accommodation conflict Stereoscopic Displays and Applications XXII; 2011: International Society for Optics and Photonics. 10.1117/12.872347 PMC3150963 21826254

[38] Kapoula Z, Adenis M-S, Lê T-T, Yang Q, Lipede G. Pictorial depth increases body sway. Psychology of Aesthetics, Creativity, and the Arts. 2011;5(2):186.

[39] Faul F, Erdfelder E, Lang A-G, Buchner A. G* Power 3: A flexible statistical power analysis program for the social, behavioral, and biomedical sciences. Behavior research methods. 2007;39(2):175–91. 17695343 10.3758/bf03193146

[40] Mergner T, Schweigart G, Maurer C, Blümle A. Human postural responses to motion of real and virtual visual environments under different support base conditions. Experimental brain research. 2005;167(4):535–56. 10.1007/s00221-005-0065-3 16132969

[41] Streepey JW, Kenyon RV, Keshner EA. Visual motion combined with base of support width reveals variable field dependency in healthy young adults. Experimental brain research. 2007;176(1):182–7. 10.1007/s00221-006-0677-2 17072608

[42] Dakin CJ, Luu BL, van den Doel K, Inglis JT, Blouin J-S. Frequency-specific modulation of vestibular-evoked sway responses in humans. Journal of neurophysiology. 2009;103(2):1048–56. 10.1152/jn.00881.2009 20032237

[43] Efron B, Tibshirani RJ. An introduction to the bootstrap: CRC press; 1994.

[44] Field A. Discopering Statistics Using SPSS, Thrid Edition London: Sage; 2009.

[45] Matheron E, Lê T-T, Yang Q, Kapoula Z. Effects of a two-diopter vertical prism on posture. Neuroscience letters. 2007;423(3):236–40. 10.1016/j.neulet.2007.07.016 17709195

[46] Kapoula Z, Leˆ T-T. Effects of distance and gaze position on postural stability in young and old subjects. Experimental brain research. 2006;173(3):438–45. 10.1007/s00221-006-0382-1 16525804

[47] Bergeron M, Lortie CL, Guitton MJ. Use of virtual reality tools for vestibular disorders rehabilitation: a comprehensive analysis. Advances in medicine. 2015;2015. 10.1155/2015/916735 PMC4590967 26556560

[48] Meldrum D, Herdman S, Vance R, Murray D, Malone K, Duffy D, et al Effectiveness of Conventional Versus Virtual Reality–Based Balance Exercises in Vestibular Rehabilitation for Unilateral Peripheral Vestibular Loss: Results of a Randomized Controlled Trial. Archives of physical medicine and rehabilitation. 2015;96(7):1319–28. e1. 10.1016/j.apmr.2015.02.032 25842051

[49] Slater M. Measuring presence: A response to the Witmer and Singer presence questionnaire. Presence. 1999;8(5):560–5.

[50] Witmer BG, Singer MJ. Measuring presence in virtual environments: A presence questionnaire. Presence. 1998;7(3):225–40.

