# Supplementary material for: Perceptual assessment of environmental stability modulates postural sway
Source: PLoS One. 2018 Nov 9;13(11):e0206218. doi: 10.1371/journal.pone.0206218 (PMC6226165; doi:10.1371/journal.pone.0206218)
Supplement: S1 File — (DOCX) [file pone.0206218.s001.docx]

Supplementary Data

# Experiment 1:

**Overall ANOVA**

Factors are:

**subFTX** – participant (random): 1-8

**cond** – viewing condition: ‘BG only’, ’FG virtual’, ’FG real’, ’BG virtual’, ’BG real’

**vis** – motion condition: BG motion vs control (no motion)

**3D02** - convergence plane: 0m and -2m conditions

| Source | Sum Sq. | d.f. | Mean Sq. | F | Prob > F |
| --- | --- | --- | --- | --- | --- |
| subFTX | 0.41976 | 10 | 0.04198 | 6.66 | 0 |
| Cond | 0.05117 | 4 | 0.01279 | 2.03 | 0.0919 |
| Vis | 0.23338 | 1 | 0.23338 | 37.01 | 0 |
| 3D02 | 0.04865 | 1 | 0.04865 | 7.71 | 0.006 |
| cond*vis | 0.03378 | 4 | 0.00845 | 1.34 | 0.2568 |
| cond*3D02 | 0.05388 | 4 | 0.01347 | 2.14 | 0.0779 |
| vis*3D02 | 0.02183 | 1 | 0.02183 | 3.46 | 0.0643 |
| Error | 1.22345 | 194 | 0.00631 |  |  |
| Total | 2.08591 | 219 |  |  |  |

Table A: ANOVA output for experiment 1.

# Experiment 1: t-tests:

Tables B and C show detailed comparisons of the VEPR component at the stimulus frequency (0.5Hz) for the motion and rest condition, the t-test result (two tailed, uncorrected) and effect size.

Table B. Lateral sway energy at 0.5Hz during visual stimulation in 0m condition

| Condition | % Energy at 0.5 Hz **Visual Motion**  Mean (SD) | | % Energy at 0.5 Hz **Rest**  Mean (SD) | | t-test results |
| --- | --- | --- | --- | --- | --- |
| **BG only** | **0.2695** | **0.06520** | **0.1642** | **0.06440** | **t_(14)_ = 3.81,**  **p = 0.0011** |
| BG virtual | 0.2016 | 0.06880 | 0.1357 | 0.04750 | t_(14)_ = 2.61,  p =  0.0166 |
| BG real | 0.2306 | 0.06190 | 0.1717 | 0.05240 | t_(14)_ = 2.41,  p = 0.0258 |
| **FG virtual** | **0.2764** | **0.04590** | **0.1689** | **0.04030** | **t_(14)_ = 5.84,**  **p < 0.0001** |
| **FG real** | **0.3327** | **0.03980** | **0.1133** | **0.03900** | **t_(14)_ = 13.06,**  **p < 0.0001** |

*Table B: Lateral Sway energy at 0.5Hz during visual stimulation and rest and comparison statistics for the target and control conditions in Experiment 1 - background at 0m. Comparisons where there are significant differences between the visual stimulation and rest condition (Bonferroni corrected alpha levels: p_corr_ < 0.005) are highlighted in bold. The correction includes all 10 tests shown in tables S2 and S3. The effect sizes (Cohen’s d) indicate the magnitude of the observed differences in mean VEPR.*

Table C. Lateral sway energy at 0.5Hz during visual stimulation in -2m condition (foreground object on projection screen, background at -2m)

| Condition | % Energy at 0.5 Hz  **Visual Motion**  Mean (SD) | | % Energy at 0.5 Hz  **Rest**  Mean (SD) | | t-test results |
| --- | --- | --- | --- | --- | --- |
| **BG only** | **0.3116** | **0.05204** | **0.1390** | **0.04940** | **t_(14)_ = 7.98,**  **p < 0.0001** |
| BG virtual | 0.2116 | 0.07790 | 0.1814 | 0.05570 | t_(14)_ = 1.05,  p = 0.31 |
| BG real | 0.2122 | 0.05550 | 0.1563 | 0.04660 | t_(14)_ = 2.55,  p = 0.019 |
| FG virtual | 0.2072 | 0.04610 | 0.1567 | 0.05149 | t_(14)_ = 2.42,  p = 0.025 |
| FG real | 0.2610 | 0.05870 | 0.2166 | 0.05344 | t_(14)_ = 1.86,  p = 0.078 |

*Table C: Lateral Sway energy at 0.5Hz during visual stimulation and rest and comparison statistics for the target and control conditions in Experiment 1 - background at 0m. Significant differences after Bonferroni correction (p_corr_ < 0.005, see table S2) are highlighted in Bold.*

Table D. Direct comparison of lateral sway energy at 0.5Hz during visual stimulation in 0m condition and in -2m conditions. VEPR components are significantly higher in the 0m condition (moving background on projection screen / without accommodation-vergence conflict.

| Condition | % Energy at 0.5 Hz  **Visual Motion at 0m**  Mean (SD) | | % Energy at 0.5 Hz  **Visual Motion at - 2m**  Mean (SD) | | t-test results |
| --- | --- | --- | --- | --- | --- |
| BG only | 0.2695 | 0.06520 | 0.3116 | 0.05204 | t_(14)_ = -1.6738,  p = 0.1097 |
| BG virtual | 0.2016 | 0.06880 | 0.2116 | 0.07790 | t_(14)_ = -0.3191,  p = 0.7529 |
| BG real | 0.2306 | 0.06190 | 0.2122 | 0.05550 | t_(14)_ = 0.7340,  p = 0.4714 |
| **FG virtual** | **0.2764** | **0.04590** | **0.2072** | **0.04610** | **t_(14)_ = 3.5280,**  **p = 0.0021** |
| **FG real** | **0.3327** | **0.03980** | **0.2610** | **0.05870** | **t_(14)_ = 3.3531,**  **p = 0.0032** |

*Table D: Lateral Sway energy at 0.5Hz during visual stimulation and rest and comparison statistics for the target and control conditions in Experiment 1, background at 0m. Comparisons where there are significant differences between the visual stimulation and rest condition. Bonferroni corrected alpha levels of p_corr_ < 0.01 are highlighted in bold. The effect sizes (Cohen’s d) indicate the magnitude of the observed differences in mean VEPR between the 0m and -2m condition.*

# Experiment 2

subFTX – participant (random)

cond – viewing condition: ‘BG only’, ’FG virtual’, ’FG real’, ’BG virtual’, ’BG real’

vis – motion condition: BG motion vs control (no motion)

stable - foreground object: teapot vs balloon

ANOVA

| Source | Sum Sq. | d.f. | Mean Sq. | F | Prob > F |
| --- | --- | --- | --- | --- | --- |
| subFTX | 0.26717 | 12 | 0.02226 | 3.02 | 0.0005 |
| Cond | 0.00744 | 4 | 0.00186 | 0.25 | 0.908 |
| Vis | 0.12915 | 1 | 0.12915 | 17.53 | 0 |
| Stable | 0.00597 | 1 | 0.00597 | 0.81 | 0.3689 |
| cond*vis | 0.04533 | 4 | 0.01133 | 1.54 | 0.1911 |
| cond*stable | 0.00856 | 4 | 0.00214 | 0.29 | 0.884 |
| vis*stable | 0.0033 | 1 | 0.0033 | 0.45 | 0.5037 |
| Error | 2.15113 | 292 | 0.00737 |  |  |
| Total | 2.61805 | 319 |  |  |  |

Table E: ANOVA output for experiment 2

Planned post-hoc comparisons excluded the four virtual foreground object conditions. As before, the tables below show uncorrected two-tailed t-test results. Conditions showing significant differences after Bonferroni correction for the six conditions tested are highlighted in bold.

Table F. Lateral sway energy at 0.5Hz during visual stimulation in teapot condition

| Condition | % Energy at 0.5 Hz Visual Motion  Mean (SD) | % Energy at 0.5 Hz Rest  Mean (SD) | t-test results | Effect size  Cohen’s d |
| --- | --- | --- | --- | --- |
| **BG only** | **0.22 (0.06)** | **0.12 (0.06)** | **t_(24)_ =**  4.2492**,**  **p = 0.0003** | **1.66** |
| BG stable | 0.15 (0.02) | 0.13 (0.04) | t_(24)_ = 1.6125,  p = 0.12 | 0.63 |
| **FG stable** | **0.25 (0.03)** | **0.13 (0.04)** | **t_(24)_ = 5.0502**  **p = 0.0001** | **3.39** |

*Table F: Lateral sway energy at 0.5Hz during visual stimulation and pause. Comparisons where there are significant differences between the visual stimulation and rest condition after Bonferroni correction ( p_corr_ < 0.016) are shown in bold. The significant VEPRs were recorded in BG only and FG real conditions. No significant VEPRs were recorded for BG real condition.*

Table G. Lateral sway energy at 0.5Hz during visual stimulation in balloon condition

| Condition | Energy @ 0.5 Hz **Vis Motion**  Mean (SD) | Energy @ 0.5 Hz **Rest**  Mean (SD) | t-test results | Effect size  Cohen’s d |
| --- | --- | --- | --- | --- |
| **BG only** | **0.17 (0.09)** | **0.09 (0.03)** | **t_(24)_ =** 3.0405**,**  **p =** 0.0056 | **1.19** |
| **BG unstable** | **0.26 (0.04)** | **0.17 (0.04)** | **t_(24)_**  5.7364 **,**  **p = 0.0001** | **2.25** |
| FG unstable | 0.21 (0.05) | 0.17 (0.05) | t_(24)_ = 2.0396  p = 0.0525 | 0.8 |

*Table G: Lateral sway energy at 0.5Hz during visual stimulation and pause. Comparisons where p values are below the Bonferroni corrected threshold of p­_min_ = 0.017 (six one-sided tests in tables F and G) differences between the visual stimulation and rest condition. Significant VEPRs were observed in the BG only condition and in the BG real condition. Significant VEPRs were not recorded in the BG real condition.*

Table H. Direct comparison for VEPRs in the motion condition for the teapot (stable) and helium balloon (unstable) as reference object.

| Condition | Teapot  Mean (SD) | Balloon  Mean (SD) | t-test results | Effect size  Cohen’s d |
| --- | --- | --- | --- | --- |
| **BG** | **0.15 (0.02)** | **0.26 (0.04)** | **t­_(14)_ = 6.9570   p < 0.0001** | **3.47** |
| **FG** | **0.25 (0.03)** | **0.21 (0.05)** | **t(24)=2.4734 ,**  **p = 0.021** | **0.97** |

*Table H: Lateral sway energy at 0.5Hz during visual stimulation in the teapot and helium balloon conditions. Comparisons where there are significant differences between the visual stimulation and rest condition. Significant VEPRs were observed in the BG real condition, they are marginally significant in the GF real condition after Bonferroni correction for two tests (p<0.025).*
